# Routine statins use is associated with less adverse outcome in patients above 70 years of age admitted to hospital with COVID-19

**DOI:** 10.1186/s12877-023-04183-8

**Published:** 2023-08-07

**Authors:** Elena Izkhakov, Yair Vilian, Assaf Buch, Veronika Denysov, Dima Namouz, Alexandra Nathan, Yona Greenman, Tomer Ziv-Baran

**Affiliations:** 1 Institute of Endocrinology, Metabolism and Hypertension, Tel Aviv Sourasky Medical Center, 6 Weizmann Street, 6423906 Tel Aviv, Israel; 2 Sackler Faculty of Medicine, Tel Aviv University, Tel Aviv, Israel; 3 Department of Epidemiology and Preventive Medicine, School of Public Health, Sackler Faculty of Medicine, Tel Aviv University, Tel Aviv, Israel

**Keywords:** Statins, COVID-19, SARS-CoV-2, Older adults, Adverse outcome

## Abstract

**Background:**

Coronavirus disease 2019 (COVID-19) caused by severe acute respiratory syndrome coronavirus 2 (SARS-CoV-2) can lead to multiorgan insufficiency and death, particularly among the older adults. Statins have been suggested as potentially protective drugs due to their pleotropic effects, but the actual benefit of statin use among the older population in this setting is not clear. This study aimed to evaluate the association between preadmission statin use and the presentation and clinical outcomes of hospitalized COVID-19 patients older than 70 years of age.

**Methods:**

A historical cohort study of all patients above 70 years of age who were hospitalized with COVID-19 infection in a large academic hospital between March and August 2020 was performed. The association between preadmission statin use and patients’ presentation and adverse outcome was studied. Adverse outcome was defined as any of the following: shock, invasive or non-invasive ventilation, organ insufficiency, myocardial infarction, cerebrovascular accident, in-hospital or 30-day post-admission mortality, hospital stay longer than the median length of stay of all COVID-19 patients, referral to nursing home or rehabilitation center.

**Results:**

Seventy-two (44%) of the 163 studied patients (median age 82 years, 45% males) had been preadmission treated with statins. The statin-treated patients (STP) had a higher prevalence of diabetes (40% vs 24%, *p* = 0.028) and cardiovascular disease (58% vs. 34%, *p* = 0.002). Seventy two percent of the STP had adverse outcome, compared to 86% of the non-STP (*p* = 0.033). After adjustment for potential confounders, prior statin use was associated with decreased risk for an adverse outcome (odds ratio = 0.4, 95% confidence interval 0.18–0.92, *p* = 0.03).

**Conclusions:**

The preadmission use of statins was associated with a lower risk of adverse outcome in older adults hospitalized with COVID-19. Continuation of statin treatment might be implemented for risk reduction of adverse outcomes in the older population in the era of new SARS-CoV-2 variants and less effective vaccines.

## Background

As of March 2022, coronavirus disease-2019 (COVID-19) caused by severe acute respiratory syndrome coronavirus 2 (SARS-CoV-2) has affected over 470,000,000 people and caused more than 6,100,000 deaths worldwide [1]. COVID-19 may lead to multiorgan insufficiency, particularly among the older population and patients with chronic conditions, such as cardiovascular disease, chronic obstructive pulmonary disease, diabetes mellitus and obesity [2–4]. Older adults are generally defined as those 70 years of age and older [5], and they have an increased risk to develop atherosclerotic cardiovascular disease. Statins are the most effective treatment for primary prevention and even more significant with regard to secondary prevention of cardiovascular disease, having been shown to decrease the risk of both cerebrovascular and coronary artery disease in older adults, similar to their effects in young populations [6, 7].

A beneficial role of statins was shown in the patients with pneumonia, other infection diseases and sepsis based upon their well-established pleotropic properties [8–10]. The association between statins and the clinical outcomes of COVID-19 patients was recently investigated in several retrospective studies, meta-analyses and a few randomized controlled trials. Most of those studies found that statin use was associated with improved clinical outcomes, while others reported no such association and even adverse outcomes among statin users [11–26]. Although some studies included stratification according to age and showed mixed results, only one study directly addressed disease outcome specifically among hospitalized and non-hospitalized older patients, while there is no reported meta-analysis on this specific population [13]. The aim of this retrospective study was to evaluate the association between preadmission statin use and the presentation and clinical outcomes of hospitalized COVID-19 patients older than 70 years of age.

## Methods

### Study design and population

A historical cohort study of all patients above 70 years of age who were hospitalized with COVID-19 infection during the first wave of the pandemic and who received the same pharmacological treatments, which included steroids, was performed between March and August 2020 at the Tel Aviv Sourasky Medical Center (TASMC). TASMC is a tertiary university-affiliated 1500-bed medical center located in central Israel. Only patients with laboratory-confirmed COVID-19 infections were included in the study. Excluded from the study were patients whose data could not be accessed, as well as subjects who had end-stage/advanced malignancy, liver cirrhosis, past single or multiple organ transplantations, and those who continued care at another acute medical center. Also excluded were patients with nosocomial COVID-19 according to European Centre for Disease Prevention and Control (CDC) (i.e., diagnosed after the 7th day of hospitalization), women who were admitted to hospital to give birth and pregnant women. The study was approved by the local ethics committee (TLV-0341–20).

### Data source, measurement and variables

All of the analyzed data were retrieved from the hospital database. Data on demographic parameters (age and gender), anthropometric measurements (weight and body mass index), first vital signs on emergency department admission (pulse, systolic blood pressure, diastolic blood pressure, body temperature and oxygen saturation), comorbidities, first blood test findings on emergency room admission and chronically used medications (angiotensin-converting enzyme inhibitors, angiotensin II receptor blockers, calcium channel blockers, beta blockers, diuretics, aspirin and insulin) were collected. Information on hypertension (HTN), diabetes mellitus (DM), cardiac disease, cerebrovascular disease (CVA/TIA), chronic renal failure (CRF), chronic obstructive pulmonary disease (COPD) or asthma, chronic liver disease and hypothyroidism was also recorded. Disease severity was determined using the NIH classification criteria [27].

Blood tests results included complete blood count, C-reactive protein (CRP), creatinine, and liver enzymes (alanine transaminase, aspartate transaminase, alkaline phosphatase and gamma glutamyl transferase). The estimated glomerular filtration rate was calculated with the CKD-EPI equation [28]. Shock, invasive or non-invasive ventilation, organ insufficiency, myocardial infarction or cerebrovascular accident during admission, in-hospital or 30-day post-admission mortality, hospital stay longer than the median length of all-adult COVID-19 patients’ stay and new referral to a nursing home or rehabilitation center were considered as an adverse outcome.

### Laboratory methods

Blood cell counts were performed with the Beckman Coulter UniCel. Blood chemistry tests were measured by ADVIA (Siemens Healthcare Diagnostics Inc., Tarrytown, NY 10591–5097 USA) as described previously [29]. Viral respiratory infection was diagnosed by polymerase chain reaction methodology.

### Statistical analysis

Sample size was calculated using a significance level of 5% and a power of 80%. We assumed that approximately 30% of the STP would have an adverse outcome compared to 55% of non-STP. In addition, a ratio of 1:1 between STP and non-STP groups was assumed, whereupon 136 individuals would be needed.

Categorical variables were described as frequency and percentage. Continuous variables were evaluated for normal distribution using histograms and Q-Q plots and reported as median and interquartile range (IQR) for readers’ ease. The Chi-square test and Fisher’s exact test were used to compare categorical variables and independents samples, and the T-test and Mann–Whitney test were applied to compare continuous variables. Due to the limited sample size and the variety of medications, the associations between medication and adverse event were included only in the univariate analysis. A multivariable logistic regression was used to identify the independent association between routine statin use and adverse outcome. The multivariable analysis included only pre-admission variables, since vital signs and blood tests at admission may represent the acute phase rather than the patient's background status. Due to the restrictions imposed by the pandemic setting, anthropometric measurements were estimated rather than precisely measured in some patients, and those parameters were not included in the multivariable analysis.

The regression included two blocks: routine statin use was entered in the first block while age, gender and all comorbidities were entered in the second block followed by the application of a backward method with the Wald test to remove variables with a *p* value > 0.1. All statistical tests were two-tailed, and *p* < 0.05 was considered statistically significant. All statistical analyses were performed with SPSS (IBM Corp. Released 2020. IBM SPSS Statistics for Windows, Version 27.0. Armonk, NY: IBM Corp.)

## Results

### Patients’ characteristics

A total of 163 patients met the inclusion criteria during the study period. The group's median age was 82 years and 73 (45%) were males. HTN was the most common comorbidity (*n* = 104, 64%), followed by cardiac disease (*n* = 73, 45%), DM (*n* = 51, 31%) and COPD/asthma (*n* = 33, 20%). The prevalence of other studied comorbidities was less than 20%. Forty one (25%) patients had an estimated glomerular filtration rate less than 45 mL/min/1.73m^2^ on admission, and the group’s median CRP was 57 mg/L. Seventy two (44%) patients used statins regularly before being admitted to the hospital, 55 (34%) used aspirin, 60 (37%) used beta blockers, 51 (31%) used calcium channel blockers and 47 (29%) used diuretics. Table 1 summarizes the patients’ characteristics.

**Table 1:** Demographic and clinical characterization of the study population – comparison between patients with and without routine statin use

**Characteristics**		**Routine statin use**		
	**All (*n* = 163)**	**No (*n* = 91)**	**Yes (*n* = 72)**	***p*-value**
Demographic parameters				
Male	73 (44.8%)	41 (45.1%)	32 (44.4%)	0.938
Age (years)	82.0 (75.4–87.8)	82.7 (75.7–89.2)	79.7 (75.0–87.0)	0.168
Anthropometric measurements				
Weight (kg)	70 (61–82)	70 (61–80)	71 (62–84)	0.518
Body mass index (kg/m^2^)	26.4 (23.4–30.3)	24.7 (22.5–28.0)	27.6 (23.8–31.6)	0.023
Vital signs on admission				
Pulse (beats per minute)	84 (72–93)	83 (72–92)	85 (73–95)	0.945
Systolic blood pressure (mm Hg)	140 (123–155)	140 (124–153)	142.5 (121–155)	0.629
Diastolic blood pressure (mm Hg)	70 (61–81)	70 (60–82)	69 (62–80)	0.970
Temperature (^o^C)	37.4 (36.8–38.0)	37.3 (36.8–38.1)	37.4 (36.9–38.0)	0.754
Oxygen saturation (%)	95 (88–97)	95 (88–97)	94 (87–97)	0.726
Comorbidity				
Hypertension	104 (63.8%)	53 (58.2%)	51 (70.8%)	0.097
Diabetes mellitus	51 (31.3%)	22 (24.2%)	29 (40.3%)	0.028
Cardiac disease	73 (44.8%)	31 (34.1%)	42 (58.3%)	0.002
Cerebrovascular disease	27 (16.6%)	12 (13.2%)	15 (20.8%)	0.192
Chronic renal failure	22 (13.5%)	10 (11%)	12 (16.7%)	0.292
COPD/asthma	33 (20.2%)	22 (24.2%)	11 (15.3%)	0.160
Chronic liver disease	0 (0%)	0 (0%)	0 (0%)	NA
Hypothyroidism	28 (17.2%)	11 (12.1%)	17 (23.6%)	0.053
Blood tests on admission				
Hemoglobin (g/dL)	12.9 (11.7–14.1)	12.9 (11.6–14.2)	12.9 (11.7–14.1)	0.719
White blood cell count (K/mcL)	6.7 (5.1–9.1)	7.5 (5.4–9.4)	6.3 (5.1–8.5)	0.130
Neutrophils				
Count (K/mcL)	4.9 (3.6–7.1)	5.4 (3.7–7.3)	4.4 (3.6–6.9)	0.108
Percent (%)	73.5 (66.4–82.9)	74.2 (67.7–83.2)	72.2 (62.2–82.5)	0.257
Lymphocytes				
Count (K/mcL)	1.0 (0.6–1.4)	1.0 (0.6–1.4)	0.9 (0.6–1.5)	0.745
Percent (%)	16.1 (9.3–22.8)	14.9 (9.1–21.0)	18.6 (9.4–25.1)	0.093
Platelet count (K/mcL)	181 (138–231)	189 (143–250)	164 (133–201)	0.013
Neutrophil-to-lymphocyte ratio	4.6 (2.8–8.8)	5.0 (3.3–9.4)	3.9 (2.5–8.4)	0.093
Platelet-to-lymphocyte ratio	178 (125–264)	192 (135–298)	167 (116–247)	0.091
C-reactive protein (mg/L)	57.2 (16.5–129.1)	62.7 (18.0–130.6)	44.0 (10.9–127.8)	0.251
Creatinine (mg/L)	0.98 (0.72–1.32)	0.97 (0.72–1.23)	1.03 (0.72–1.40)	0.280
Estimated glomerular filtration rate (mL/min/1.73m^2^)	68.6 (44.8–86.3)	69.9 (46.6–85.7)	66 (42.7–86.9)	0.430
Alanine transaminase (U/L)	23 (15–31)	21 (15.5–26.5)	25.5 (15–39)	0.148
Aspartate transaminase (U/L)	30 (24–46)	29 (22–45)	32 (25–48)	0.405
Alkaline phosphatase (U/L)	65 (53–85)	70 (55–88)	60 (50–84)	0.171
Gamma glutamyl transferase (U/L)	26 (18–47)	28 (18–45)	24 (17–49)	0.840
Chronic medication				
Angiotensin-converting enzyme inhibitors, n (%)	27 (16.6%)	10 (11%)	17 (23.6%)	0.031
Angiotensin II receptor blockers, n (%)	40 (24.5%)	16 (17.6%)	24 (33.3%)	0.020
Calcium channel blockers, n (%)	51 (31.3%)	24 (26.4%)	27 (37.5%)	0.128
Beta blockers, n (%)	60 (36.8%)	27 (29.7%)	33 (45.8%)	0.034
Diuretics, n (%)	47 (28.8%)	22 (24.2%)	25 (34.7%)	0.140
Aspirin, n (%)	55 (33.7%)	16 (17.6%)	39 (54.2%)	< 0.001
Insulin, n (%)	7 (4.3%)	5 (5.5%)	2 (2.8%)	0.466

Continuous variables reported as median and interquartile range (IQR)

Age and gender distribution of patients who were taking statins regularly (STP) were not significantly different from those who did not use statins (NSTP), while DM and cardiovascular disease were more common among the STP (40% vs 24% and 58% vs. 34%, respectfully). Vital signs, blood test results and the severity of COVID-19 at emergency department admission were not statistically different between the two groups. Angiotensin-converting enzyme inhibitors, angiotensin II receptor blockers, calcium channel blockers, beta blockers, diuretics and aspirin were more commonly used by patients who also used statins on a regular basis. Table 1 compares patients’ characteristics in the two groups.

### Adverse outcome

**Fig. 1:**
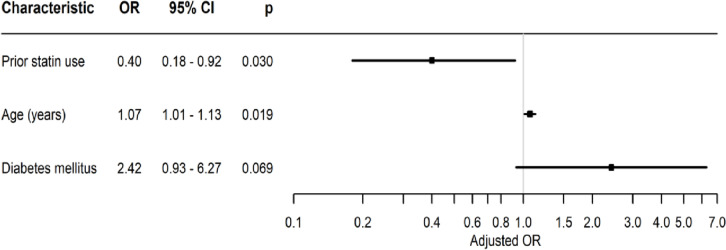
A Forest plot summarizing the multivariable analysis of potential predictors to adverse outcome in patients above 70 years of age hospitalized with COVID-19 infection

In total, 130 patients sustained an adverse outcome during hospitalization, including 10 patients with shock, 111 who were ventilated (29 invasive and 107 non-invasive), 54 with organ insufficiency and 6 with myocardial infarction or a cerebrovascular accident. Forty-seven patients died in the hospital and another 2 patients died during 30 days post-admission. In addition, 111 were hospitalized longer than the median length of stay of all-age COVID-19 patients (5 days), 7 were referred to nursing homes and 7 were referred to rehabilitation centers. Taken together, these findings revealed that 72% of the STP had an adverse outcome compared to 86% of NSTP (*p* = 0.033). The patients who sustained adverse events were older (median 82.7 vs 76.1 years, *p* = 0.011), had lower hemoglobin levels (12.5 vs. 13.8 g/dL, *p* = 0.005) and higher inflammatory biomarkers on admission (median WBC 7.5 vs. 6.1 K/mcL, *p* = 0.002, median CRP 67.1 vs 10.7, *p* < 0.001). Comorbidities and chronic treatments with medications other than statins were not significantly different between the patients with and those without an adverse outcome. Table 2 presents the crude association between an adverse outcome and patient characteristics. After controlling for potential confounders, prior statin use was associated with a decreased risk for an adverse outcome (odds ratio [OR] = 0.4, *p* = 0.03), while older age (OR = 1.07, *p* = 0.019) was associated with an increased risk (Fig. 1).

**Table 2:** Comparison of characteristics between patients with and without adverse outcome

Characteristics	No (*n* = 33)	Yes (*n* = 130)	p
Demographic parameters			
Male, n (%)	13 (39.4%)	60 (46.2%)	0.486
Age (years)	76.1 (72.8–83.9)	82.7 (76.5–88.5)	0.011
Anthropometric measurement			
Weight (kg)	62 (55–77)	72 (65–84.5)	0.043
Body mass index (kg/m^2^)	26.7 (22.9–28.0)	26.3 (23.4–31.2)	0.197
Vital signs at admission			
Pulse (beats per minute)	73 (68–85)	86 (76–96)	0.002
Systolic blood pressure (mm Hg)	149 (131–157)	139 (122–154)	0.120
Diastolic blood pressure (mm Hg)	70 (62–81.5)	70 (60–81)	0.976
Temperature (^o^C)	37.1 (36.8–37.6)	37.4 (36.9–38.1)	0.013
Saturation (%)	96 (95–98)	93 (85–97)	0.001
Comorbidity			
Hypertension, n (%)	22 (66.7%)	82 (63.1%)	0.702
Diabetes mellitus, n (%)	7 (21.2%)	44 (33.8%)	0.162
Cardiac disease, n (%)	17 (51.5%)	56 (43.1%)	0.384
Cerebrovascular disease, n (%)	7 (21.2%)	20 (15.4%)	0.421
Chronic renal failure, n (%)	3 (9.1%)	19 (14.6%)	0.571
COPD/asthma, n (%)	4 (12.1%)	29 (22.3%)	0.193
Hypothyroidism, n (%)	6 (18.2%)	22 (16.9%)	0.864
Blood test results at admission			
Hemoglobin (g/dL)	13.8 (12.9–14.4)	12.5 (11.4–14.1)	0.005
White blood cells count (K/mcL)	6.1 (4.4–6.8)	7.5 (5.1–9.8)	0.002
Neutrophils			
Count (K/mcL)	4.2 (2.9–4.8)	5.5 (3.7–7.8)	0.001
Percent (%)	67.1 (62.1–74.4)	75.4 (66.9–83.7)	0.009
Lymphocytes			
Count (K/mcL)	1.1 (0.7–1.6)	1.0 (0.6–1.4)	0.301
Percent (%)	22.0 (13.7–26.3)	14.9 (9.1–21.2)	0.003
Platelet count (K/mcL)	149 (132–181)	187 (142–239)	0.004
Neutrophil-to-lymphocyte ratio	3.0 (2.4–5.9)	5.1 (3.1–9.4)	0.002
Platelet-to-lymphocyte ratio	141 (108–223)	193 (135–298)	0.007
C-reactive protein (mg/L)	10.7 (4.0–41.2)	67.1 (27.9–138.2)	< 0.001
Creatinine (mg/L)	0.97 (0.72–1.20)	0.99 (0.72–1.48)	0.506
Estimated glomerular filtration rate (mL/min/1.73m^2^)	71.7 (58–86.4)	67.2 (42.3–86.1)	0.377
Alanine transaminase (U/L)	24 (14–30)	23 (16–32)	0.905
Aspartate transaminase (U/L)	31 (23–39)	30 (24–49)	0.637
Alkaline phosphatase (U/L)	58 (50–78)	68 (54–88)	0.101
Gamma glutamyl transferase (U/L)	22 (15–39)	28 (19–49)	0.060
Chronic medication			
Statins, n (%)	20 (60.6%)	52 (40.0%)	0.033
Angiotensin-converting enzyme inhibitors, n (%)	8 (24.2%)	19 (14.6%)	0.184
Angiotensin II receptor blockers, n (%)	10 (30.3%)	30 (23.1%)	0.389
Calcium channel blockers, n (%)	6 (18.2%)	45 (34.6%)	0.069
Beta blockers, n (%)	14 (42.4%)	46 (35.4%)	0.454
Diuretics, n (%)	8 (24.2%)	39 (30.0%)	0.514
Aspirin, n (%)	14 (42.4%)	41 (31.5%)	0.238
Insulin, n (%)	1 (3.0%)	6 (4.6%)	> 0.999

Continuous variables reported as median and interquartile range (IQR)

## Discussion

COVID-19 caused by SARS-CoV-2 first appeared in late 2019 and spread rapidly, leading to a global pandemic. The older population has been reported to be at increased risk for serious outcomes in many studies. The newly developed vaccines have led to a significant reduction in morbidity and mortality, however, the appearance of the new variants elicited a significant decrease in vaccine efficacy. Statin therapy is common in the older population, especially among those with major illnesses associated with poor disease outcomes. This addition of new therapies designed to contend with an unprecedented milieu emphasizes the importance of examining the association between routine statin use and poor outcome, specifically among older COVID-19 patients. This study, therefore, aimed to evaluate the association between preadmission statin use and the presentation and clinical outcomes of hospitalized COVID-19 patients older than 70 years of age.

The results of this retrospective study suggest that although there were no significant clinical differences between the STP group and the NSTP group with regard to the severity of COVID-19 at presentation, preadmission statin use was associated with a decreased risk for an adverse outcome (OR = 0.4, 95% confidence interval [CI] 0.18–0.92, *p* = 0.03).

As expected, more STP presented with DM compared to NSTP (40% vs 20%, respectively) as well as with cardiovascular disease (58% vs 34%). Memel et al. described higher rates of HTN, DM, coronary artery disease, heart failure, and chronic kidney disease among 43.1% of their STP who were > 65 years of age [17]. El-Solh et al. analyzed data from the USA Department of Veterans Affairs (not all hospitalized patients): 50.2% were STP who were older than the NSTP group (68.2 vs. 58.9 years, respectively, *p* < 0.001) and had higher rates of HTN, DM, cardiac diseases, and chronic kidney disease [19]. De Spiegeleer et al. investigated the effects of statins on clinical outcomes of COVID-19 infection among 154 nursing home residents with a mean age of 86 years [13]. Those authors found an association between statin treatment and asymptomatic COVID-19 infection (OR 2.65; CI 1.13–6.68) among that older population. The statin-treated ones tended to have less serious clinical outcomes but not to a level of significance (OR 0.75; CI 0.24–1.87).

There are several explanations for the positive effect of statins on clinical outcome after COVID-19 infection. First, they show immune modulation and counteraction against the inflammatory response and the cytokine storm leading to the acute respiratory distress syndrome often present in viral infections. Second, stabilization by statins of the product of the MYD88 gene that regularly activates NF-κB resulting in mitigation of the inflammatory response has been reported in animal models [30–33]. Third, SARS-CoV-2 invades cells through the angiotensin-converting enzyme 2 receptor which is upregulated by statins. Fourth, statins have a role in preventing endothelial dysfunction and hypercoagulability, which are major contributors to the pathogenesis of COVID-19 [11, 12, 34]. Lastly, statins have the potential of acting against the negative effect of some of the other COVID-19 drugs on the lipid profile [35, 36].

The major strength of this study is the use of systematic electronic medical records of preadmission STP older than 70 years of age at the time of admission for COVID-19. The study was designed to include all consecutive patients that met the inclusion and exclusion criteria, thus, making selection bias unlikely to occur. All studied outcomes were fully recorded in the patients' records, and disease severity was based upon updated NIH classification [27].

Our study has a several limitations that bear mention. First, it includes a retrospective cohort of patients treated in a single medical center. Second, there is the possibility that some of the same patients were later readmitted to a different hospital, resulting in the loss of final follow-up of the patients in our database. Since the number of those latter patients is negligible, however, the influence of that caveat on the study results is likely to be small. Finally, our cohort of patients was recruited from the beginning of the pandemic period, before drugs intended to directly treat the COVID-19 patients were offered and before vaccines were available. However, those drawbacks did not prevent this cohort of patients to enable us to evaluate the association between statin use and COVID-19 severity among the older population.

In conclusion, the results of this retrospective study showed significant protective effects of statin use on adverse outcomes among older adult patients hospitalized for COVID-19, and demonstrated the importance of continuing chronic statin therapy.

## Data Availability

The data that support the findings of this study are available from database of the Tel Aviv Sourasky Medical Center, but restrictions apply to the availability of these data, which were used under license for the current study, and so are not publicly available. Data are however available from the authors upon reasonable request and with permission of the Tel Aviv Sourasky Medical Center.

## Abbreviations

COVID-19: Coronavirus disease 2019
SARS-CoV-2: Severe acute respiratory syndrome coronavirus 2
STP: Statin-treated patients
NSTP: Non-statin-treated patients
